# Probiotic Effects of *Arthrobacter nicotianae* and *Bacillus cereus* on the Growth, Health, and Microbiota of Red Tilapia (*Oreochromis* sp.)

**DOI:** 10.1155/anu/6074225

**Published:** 2025-05-01

**Authors:** Remy Ntakirutimana, K. M. Mujeeb Rahiman, K. V. Neethu

**Affiliations:** ^1^Centre de Recherche en Sciences Naturelles et de l'Environnement (CRSNE), University of Burundi, Bujumbura, Burundi; ^2^School of Industrial Fisheries, Cochin University of Science and Technology, Cochin, Kerala, India; ^3^Department of Marine Biology, Microbiology and Biochemistry, Cochin University of Science and Technology, Cochin, Kerala, India

**Keywords:** *Arthrobacter nicotianae*, *Bacillus cereus*, hematology, intestine histology, microbiota, probiotics, red tilapia

## Abstract

This study evaluated the effects of a commercial probiotic containing *Arthrobacter nicotianae* and *Bacillus cereus* on the growth performance, intestinal histological structure, body composition, hematology, and microbiota of red tilapia. Fingerlings were fed four different diets: a control diet (Pd0) and three diets (Pd1, Pd2, and Pd3) containing 15, 20, and 40 mL of probiotics/kg, respectively, for 12 weeks. Probiotic supplementation had no significant effect on water quality parameters. Compared with the control diet, all the probiotic diets improved growth performance, with greater final body weight (FBW), net weight gain (NWG), weight gain, average daily weight gain (ADWG), specific growth rate (SGR), and feed conversion efficiency (FCE). The feed conversion ratio (FCR) was lower in all probiotic-treated fish compared to control. The survival rate was also higher in the probiotic groups, though the difference was not significant. There was no significant difference in crude ash or lipid contents. However, protein content was significantly higher in Pd2 and Pd3, while moisture content (MC) was significantly higher in Pd3 than in the control group. Histological examination revealed increased villi length and width, being significantly higher in Pd2 and Pd3, while significantly greater muscular thickness and intestinal diameter were observed in Pd3-treated fish. These values increased with probiotic dose. The intestinal total viable count (TVC) was the highest in Pd2 and the lowest in the control group. The water TVC was the highest in Pd3 and the lowest in Pd0. The number of *Bacillus* spp. in the intestine and culture water increased with probiotic dose, while intestinal and culture water *Vibrio* counts decreased. Hematological analysis showed significant increases in red blood cell (RBC) count, hematocrit, mean corpuscular hemoglobin concentration (MCHC), and hemoglobin (Hb) in the treated groups compared with the control. The incorporation of *A. nicotianae* and *B. cereus* at 40 mL/kg in red tilapia diets improved growth performance, intestinal health, and general welfare.

## 1. Introduction

In recent decades, aquaculture has become a crucial sector in meeting the global demand for seafood, with tilapia being one of the most widely cultivated species in the world [1–3]. The tilapia farming industry contributes significantly to food security, economic stability, and livelihoods worldwide. This species is a sustainable and efficient choice for aquafarmers due to its rapid growth rate and adaptability to various habitats, meeting the increasing demand for seafood [1, 2, 4, 5]. Sustainable aquaculture production relies on maximizing growth performance, fish health, and flesh quality, which can be enhanced through specially designed diets [6–8]. The intensification of aquaculture has raised concerns about the increased risk of pathogenic diseases, particularly in fish farming [1]. For years, aquafarmers have relied heavily on antibiotics to combat these diseases [9–11]. However, excessive antibiotic use has led to severe environmental and human health consequences, including antimicrobial resistance and chemical contamination [9, 12, 13]. To address these challenges, researchers and aquafarmers have explored alternative approaches to disease prevention and treatment, with probiotics emerging as promising bioenhancers [14, 15]. These beneficial microorganisms improve host health, enhance nutrient utilization, and inhibit pathogen colonization [16–18]. The use of probiotics in fish farming supports sustainable production by reducing disease risk, optimizing growth, and improving fish quality [11, 16, 19]. Probiotics include bacteria, yeasts, and microalgae, each offering specific benefits such as improved digestion, nutrient absorption, pathogen resistance, immune system modulation, and enhanced water quality [20–22]. Among the commonly used probiotic genera in aquaculture are *Bacillus*, lactic acid bacteria (*LAB*), *Bifidobacteria*, *Pseudomonas*, *Arthrobacter*, and *Enterococcus* [16, 20, 23]. The genus *Arthrobacter* has been recognized for its probiotic potential, particularly in shellfish, due to its ability to produce antipathogenic substances [20]. For instance, *Arthrobacter* XE-7 improved intestinal bacterial counts and reduced nitrogen and nitrate–nitrogen concentrations in shrimp larval culture [24]. Additionally, *Arthrobacter* XE-7 provided immunization and protection against *Vibrio parahaemolyticus* in Pacific white shrimp by enhancing hemocyte counts, phagocytosis, respiratory activity, and serum phenol oxidase activity while reducing mortality [25]. *Bacillus* species are widely used as probiotics due to their gram-positive, catalase-positive, facultative anaerobic, and spore-forming characteristics, which ensure long-term viability and stability in aquatic environments [26–28]. *Bacillus subtilis* and *Bacillus licheniformis* have been shown to enhance growth performance, gut microbiota, and innate immunity in Nile tilapia [29]. Similarly, *B. subtilis* improves growth, immunity, intestinal histology, and disease resistance gene expression in tilapia [30, 31]. The use of multistrain *Bacillus* concentrates has also been reported to improve water quality, growth parameters, intestinal microbiota, hematology, and intestinal histology in Nile tilapia [32]. To the best of our knowledge, no study has investigated the application of a probiotic containing *Arthrobacter* and *Bacillus* in red tilapia. Thus, the current study aimed to evaluate the effects of different doses of commercial probiotic products containing *B. cereus* and *A. nicotianae* to identify an efficient dosage that can ensure better growth, survival, intestinal histology, intestinal microbiota, hematological parameters, and biochemical body composition in red tilapia over 12 weeks.

## 2. Materials and Methods

### 2.1. Experimental Diet and Probiotics

The ingredients used in the feed formulation as well as the nutritional composition of the feed are presented in Table 1. The probiotic Enterotrophotic, which is a blend of *B. cereus* sensulato (MCCB 101) (GenBank EF 062509) and *A. nicotianae* (MCCB 104) (GenBank EU402968), was purchased from the National Centre for Aquatic Animal Health (NCAAH), CUSAT. Fish meal, soybean flour, wheat flour, rice bran flour, and cassava flour were mixed and homogenized in a mixer–blender. The mixture was placed on a tray and mixed thoroughly with warm water, and multivitamins, minerals, and sunflower oil were systematically added. After cooling, the paste formed was passed through an extruder to form pellets. The pellets were dried in an oven at 55°C until apparent dryness. The dried pellets were stored in a plastic bottle at room temperature in a cool and dry place. The biochemical composition of the dried pellets was analyzed following standardized methods [33]. Moisture content (MC) was determined by oven drying method, ash content by muffle furnace incineration at 550°C, crude protein (CP) content using the Kjeldahl method, crude lipid content by Soxhlet extraction method, and crude fiber by Muslin cloth method. Carbohydrates were estimated using the difference method, and gross energy is estimated using Atwater factor method. In accordance with the manufacturer's instructions, a precise amount of probiotics was added to the pellets 30 min before feeding. On the basis of the probiotic concentrations expressed in milliliters per kilogram of food, four diets were developed: Pd0 (control) did not receive any probiotic supplementation and Pd1, Pd2, and Pd3 received 15, 20, and 40 mL/kg probiotics, respectively. The quantity of food was adjusted to the weight of the fish fortnightly.

### 2.2. Fish Handling and Experimental Design

Red tilapia fingerlings were sourced from a local fish farmer in Angamally, Ernakulam, Kerala, and acclimated for 2 weeks. During this period, they were fed a control diet to adapt to the experimental conditions. The experiment was conducted in an indoor facility using 12 plastic tubs (~65 cm in diameter and 30.1 cm in height), each with a capacity of 100 L and filled with 75 L of water. The system functioned as a closed indoor setup with daily water exchange. Specifically, 20% of the water was removed along with waste and replaced with fresh water to maintain optimal water quality. Each tub was equipped with an aeration system to ensure adequate oxygen levels. A total of 144 fingerlings were randomly distributed at a stocking density of 12 fish per tub. A completely randomized design was implemented, consisting of four dietary treatments, each replicated three times. Fish were fed at a rate of 5% of their body weight per day, divided into three feedings, for a duration of 12 weeks.

### 2.3. Water Quality Assessment

Throughout the study period, water samples were collected from each experimental tub, and alkalinity, carbonate, bicarbonate, hydroxide, calcium, magnesium, total hardness, nitrite, sulfide, salinity, dissolved oxygen (DO), and ammonia-N were measured fortnightly, while water temperature and pH were recorded daily. The pH was measured using an electronic pH meter, and the temperature was recorded with a probe thermometer. DO concentration was determined by the Winkler method, ammonia concentration by the phenol-hypochlorite method, and nitrite concentration using the colorimetric method according to the American Public Health Association standards [34]. Alkalinity, carbonate, bicarbonate, and hydroxide were analyzed using acid titration. Calcium and magnesium concentrations, as well as total hardness, were determined using the EDTA titrimetric method. Sulfide levels were measured using the methylene blue method, and salinity was determined with a refractometer.

### 2.4. Growth-Related Parameters, Survival, and Feed Utilization

The measurements taken at the beginning and end of the feeding trial were used to calculate the production parameters on the basis of the initial body weight (IBW) and final body weight (FBW). A precision electric balance was used to determine the weight of each individual in each treatment. The following parameters were calculated: survival rate, net weight gain (NWG), percentage weight gain (PWG), average daily weight gain (ADWG), specific growth rate (SGR), feed conversion ratio (FCR), and feed conversion efficiency (FCE). They were calculated using the following formulas [30, 31, 35]:

Survival rate = final number × 100/initial number,

NWG (g) = final weight − initial weight,

PWG (%) = (mean final weight − mean initial weight) × 100/mean initial weight,

ADWG (g/fish/day) = (final weight − initial weight)/trial duration (days),

SGR (%/day) = (ln (final weight) − ln (initial weight)) × 100/trial duration (days),

FCR = total dry feed intake (g)/total fish weight gain (g),

FCE = total fish weight gain (g)/total dry feed consumed (g).

### 2.5. Body Biochemical Composition

At the end of the experimental period, three fish from each tank were taken as samples. The animals were euthanized using an overdose (250 µL/L) of clove oil anesthesia [36] and then dissected under sterile conditions. Their intestines were directly used for microbiological and histological analyses, while the remaining carcasses were stored at −20°C until further analysis. The biochemical composition of the fish muscle, including moisture, CP, crude lipid, and ash content, was analyzed following the standardized methods [33]. MC was determined by the oven drying method, ash content by muffle furnace incineration at 550°C, CP content using the Kjeldahl method, and crude lipid content by Soxhlet extraction method.

### 2.6. Bacterial Counts

In this study, the total viable count (TVC), *Bacillus* count, and *Vibrio* count were assessed. Water samples were collected from each tank for microbial analysis. The intestines, collected as described above, were homogenized, vortexed, and preserved in sterile saline solution at 4°C until analysis. Plate count agar (Standard Methods Agar) was used to determine the total bacterial count, thiosulfate–citrate–bile–sucrose (TCBS) agar was used for *Vibrio* count, and *Bacillus* agar was used for *Bacillus* count. A 200-µL (0.2 mL) aliquot of the diluted samples was inoculated in triplicate and incubated at 37°C for 24–48 h. After incubation, colonies were counted on plates containing 30–300 colonies, following the method described by Rahiman et al. [37].

### 2.7. Intestinal Histology Examination

Three fish per group were euthanized by an overdose of clove oil and then dissected to extract the intestines. For histological analysis, the proximal portion of each extracted intestine was immersed in 10% formalin immediately after dissection for fixation. The fixed tissues were rinsed with running tap water for 24 h. The tissues were then gradually passed through a series of alcohol solutions, namely, 70%, 80%, 90%, and 100% ethanol. The tissues were then cleaned by dipping them in xylene to make them transparent and facilitate embedding. The released tissues were embedded in paraffin wax to provide structural support during the cutting process. Transverse thin sections (5 µm thick) of the embedded tissues were made using a rotary microtome (Leica RM2125 RTS: Leica Biosystems). The sections were floated in a warm water bath to flatten them and collected on glass slides. The slides were completely dried at room temperature. Hematoxylin and eosin (H&E) staining was used to stain the sections for histological examination, and slides were coverslipped after staining. Images of the prepared slides were obtained under an epifluorescence microscope (Leica DM6 B) coupled to a digital camera (Leica DFC450 C). Villi height, villi width, muscular thickness, and the section diameter were measured using the Leica Application Suite software [38].

### 2.8. Hematological Parameters

For each tank, three fish were selected as samples and anesthetized via clove oil. Using a needle and an EDTA-coated syringe, blood was collected from the caudal vein [39]. The complete blood count (CBC) test was performed via electrical impedance; volume, conductivity, and scatter (VCS); and photometry. The hematological parameters measured were as follows: red blood cell (RBC) count, white blood cell (WBC) count, mean corpuscular volume (MCV), hemoglobin (Hb), packed cell volume (PCV/hematocrit), mean corpuscular hemoglobin (MCH), mean corpuscular hemoglobin concentration (MCHC), red cell distribution width (RDW), thrombocyte (platelet) count, mean platelet volume (MPV), and differential leucocyte count (neutrophil, lymphocytes, monocytes, eosinophils, and basophils).

### 2.9. Statistical Analysis

After testing for normality, a one-way ANOVA was performed to evaluate the effect of probiotic dosage on the studied parameters. Tukey's post hoc test was used to compare mean differences at a 5% significance level. Results are presented as means ± standard deviations. This statistical analysis was carried out using IBM SPSS version 25 software.

## 3. Results

### 3.1. Water Quality Parameters

The results of the water quality analysis are summarized in Table 2. There were no significant differences (*p* > 0.05) in the physicochemical parameters of the culture water among the treatments. The temperature remained at ~27°C. The pH was also stable, with values very close to 7. The alkalinity showed some variation that was not statistically significant, with a large value observed for Pd2 and a minimum value observed for Pd0. The sulfide levels were below detectable limits (BDLs) for all the treatments. The DO level was ~6 in all the replicates. Overall, the results indicate that the different dosages of the probiotic had no significant effect on the different physicochemical parameters of the culture water.

### 3.2. Growth-Related Parameters, Survival, and Feed Utilization

The results of the analyses of growth, survival, and feed utilization parameters in red tilapia fed different probiotic levels during the experimental period are presented in Table 3. Except for IBW and survival rate, all other growth and feed utilization parameters showed significant differences (*p*  < 0.05) among treatments. The FBW increased with probiotic dosage, with the highest value in Pd3 and the lowest in Pd0. The NWG (and weight gain) followed a similar trend, with Pd3 showing the highest value and Pd0 the lowest. Other parameters, including ADWG, SGR, and FCE, also improved with increasing probiotic levels. In contrast, the FCR showed an opposite trend, decreasing as probiotic dosage increased, with the highest value in Pd0 and the lowest in Pd3.

### 3.3. Fish Body Biochemical Composition

Data in Table 4 show the statistical results of the proximate composition of the fish muscle after the feeding trial. There was no significant difference (*p*  > 0.05) in ash composition among the different treatments, with values between 3.85 ± 1.23 and 4.79 ± 0.18%. The crude lipid also did not significantly differ (*p*  > 0.05) among the treatments. The values are between 2.03 ± 0.71 and 2.85 ± 0.42% of the crude lipid content. However, both the CP and MC significantly varied (*p*  < 0.05) among the treatments. The highest values of CP were observed for both Pd3 and Pd2, with values of 85.79 ± 1.75 and 85.40 ± 2.0%, respectively, followed by Pd1, with 83.66 ± 0.58%. The lowest CP (78.37 ± 3.71%) was observed for Pd0. The MC decreased with increasing probiotic dosage, with the highest value (75.13 ± 1.63%) observed in Pd0 and the lowest value (72.40 ± 1.73) observed in Pd3.

### 3.4. Microbial Composition

**Figure 1:**
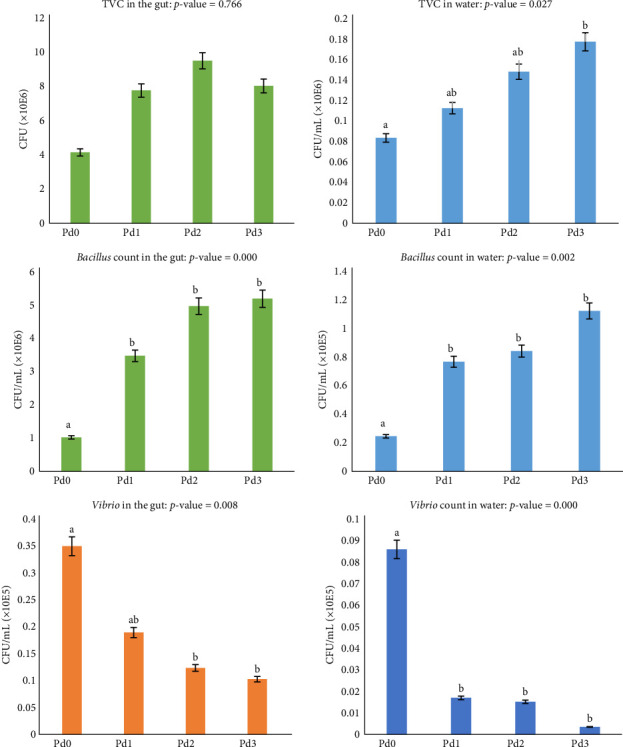
Bacterial loads in both the intestine and water after the experimental trial. CFU, colony-forming unit; TVC, total viable count. Values of bars with the same superscript letter in each chart are not significantly different.

The microbial compositions of the fish gut and rearing water for the TVC, *Bacillus* count, and *Vibrio* count are shown in Figure 1. The analysis of plate culture on plate count agar revealed a significant difference (*p* < 0.05) in TVC from the rearing water but not from the fish gut (*p* > 0.05). The highest TVC from the water samples was observed for Pd3, whereas the lowest TVC was observed in the samples from Pd0. There was a significant difference in the *Bacillus* count and *Vibrio* count from both the rearing water and the fish gut (*p* < 0.05). A high *Bacillus* count was found for Pd3, followed by Pd2 and Pd1, and the lowest value was observed in Pd0. For the *Vibrio* count, the highest value was observed in Pd0, and the value decreased with increasing probiotic dosage in both the gut and water samples.

### 3.5. Hematological Features

**Figure 2:**
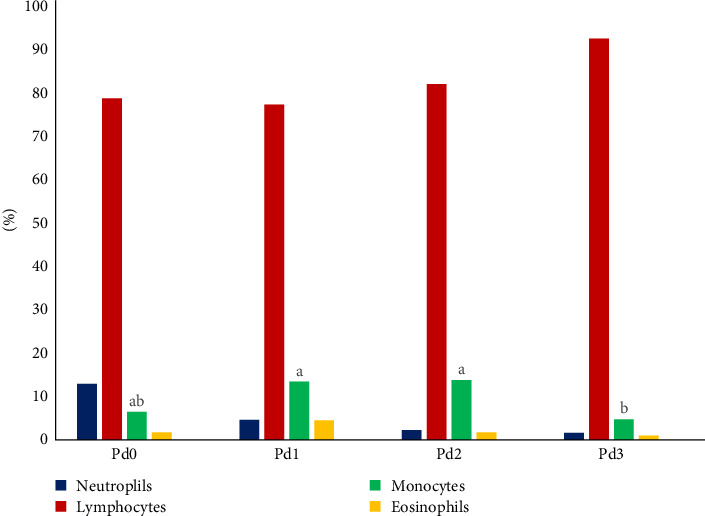
White blood cell (WBC) differential counts in red tilapia fed by different dosages of probiotics. Values of bars with the same superscript letter are not significantly different.

The results of the hematological analysis of the fish blood collected from the treatment groups are shown in Table 5, as well as in Figure 2, which shows the details of the differential WBC counts. Significant differences (*p* < 0.05) in Hb, PCV, MCHC, and monocyte levels were detected. The highest value of Hb was found in Pd2, followed by Pd3 and Pd1, while Pd0 presented the lowest value. Pd3 had the highest hematocrit value, followed by Pd1 and Pd2, and Pd0 was in the last position. Pd2 presented the highest value of MCHC, followed by Pd0 and Pd1, whereas Pd3 presented the lowest value. The monocyte count was greater for Pd2 and Pd1, followed by Pd0 and finally Pd3. There was no significant difference (*p* > 0.05) in RBC, MCH, RDW, WBC, neutrophil, lymphocyte, eosinophil, and basophil counts, platelet count, or MPV. For WBC differential counts, Figure 2 shows that lymphocytes were dominant in fish blood from all the treatment groups, followed by neutrophils from the Pd0 group and monocytes from the Pd1, Pd2, and Pd3 groups, whereas basophils were completely nonexistent in the fish blood from all the treatment groups.

### 3.6. Intestinal Histomorphology

**Figure 3:**
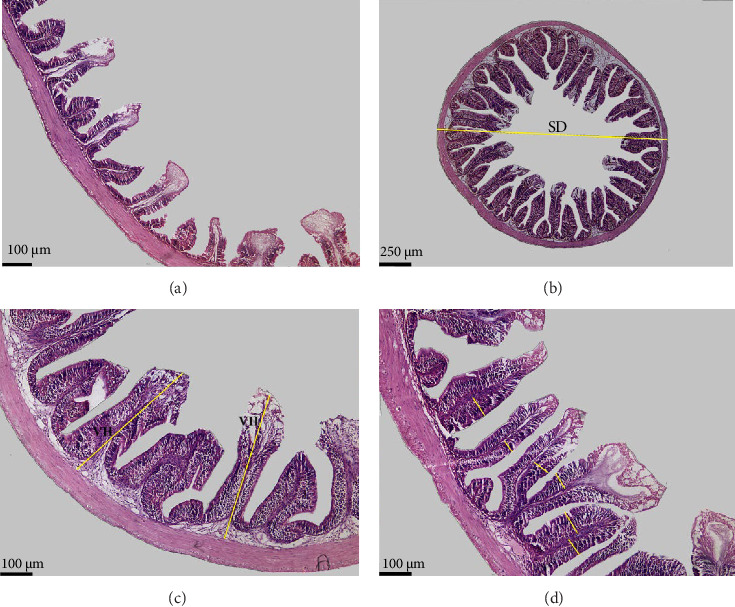
Intestine histomorphological structure of red tilapia fed different levels of probiotics. (A) showed the low villi density and height of Pd0; (B) exemplified the section diameter measurement of Pd1; (C) illustrated villi height measurement in Pd2; and (D) illustrated the measurement of the villi width in Pd3.

Data in Table 6 and Figure 3 show the images and values of the measured parameters of the histomorphological structure of the intestine. The results of the analysis of the height, width of the villi, the muscular thickness, and the diameter of the circular sections of the intestines revealed significant differences among the different groups (*p*  < 0.05). The villus height and width increased with increasing probiotic concentration, where Pd3 presented the highest values and Pd0 the lowest values. Pd3 also presented high values of muscular thickness, followed by Pd2, whereas Pd1 presented the lowest value.

## 4. Discussion

Aquafarmers and researchers around the world are still concerned about the fight against bacterial infectious diseases in fish and seafood [40]. This is because the most widely used method, the use of antibiotics, has negative effects on human health, particularly the environment and biodiversity [12, 13]. As one of the most widely farmed species, tilapia is susceptible to bacterial infectious diseases, which can have a variety of effects, ranging from decreased yield due to poor health and growth inhibition to complete losses due to mass mortality in fish and seafood [41–43]. It is still important to identify the most environmentally friendly and sustainable substitutes for antibiotics and other chemicals that the aquaculture sector typically uses [19, 44, 45]. Since probiotics are among the most promising treatments in this area [37], the effects of the commercial probiotic Enterotrophotic, which contains *B. cereus* and *A. nicotianae*, on red tilapia were assessed in this study. Changes in growth performance, the histomorphological structure of the intestines, body composition, hematological parameters, water quality, and the microbiota were assessed. Compared with those in the control diet (Pd0), the growth performance of red tilapia significantly improved in terms of weight gain and SGRs at Pd1, Pd2, and Pd3. These findings indicate that *B. cereus* and *A. nicotianae* may affect the growth performance of red tilapia, a hypothesis supported by the lowest FCR and high FCE in the probiotic-treated groups (Table 3). Similar results were reported for red tilapia fed diets supplemented with probiotics and enzymes [35]. In addition, Tabassum et al. [32] and Tachibana et al. [29] reported that growth parameters were improved by probiotics containing *Bacillus* sp. These outcomes can be attributed to the different characteristics of *Bacillus* and *Arthrobacter*. In fact, *Arthrobacter* has been described as having antimicrobial and immune system-improving properties [25], and *Bacillus* species play important roles in immune stimulation, the production of antimicrobial compounds, competitive exclusion, and the production of digestive enzymes that improve digestion and nutrient absorption [27]. Hence, the use of probiotics has a positive effect on the growth performance of fish, improvements that might be related to the enhancement of nutrient absorption, digestion, and disease protection through probiotics, improving the overall metabolic and health efficiency of fish.

There were no significant differences in any of the water quality parameters among the various treatment groups, which further signifies that the addition of probiotics did not deteriorate the rearing water. Some similar studies [35, 39, 46] also did not identify any impact of probiotics on water quality. However, some research on probiotics in various farming systems has revealed significant differences in water quality. When water is constant, as in continuous recirculation [47, 48] or biofloc systems [49–51], especially in natural environments such as ponds and cage farming, water parameters vary significantly [52–54]. Nonetheless, it is typical for conditions to remain favorable and similar in all treatments in an indoor experimental setting where water is routinely diluted and monitored along with the regular removal of all organic matter. Biological and chemical reactions that can alter water parameters are unlikely to occur under these circumstances [55, 56]. Hence, these findings are important because probiotics can be used without having detrimental effects on the aquatic environment to support sustainable aquaculture practices.

Compared with those from the control group, the histological structure of the intestines from the fish fed the probiotic-supplemented diets was greater and more integral. Pd3 and Pd2 presented significantly greater values of villi height and width, whereas the values of Pd1 were statistically close to those of Pd0. Muscular thickness was very important for Pd3, and Pd1 presented the lowest value. The improvements in the structural integrity of the intestine are important because they increase the surface area for nutrient absorption, thereby promoting better growth and health. Villus size and density are very important, as their increase results in a large surface area for nutrient absorption [19, 57]. Muscle thickness allows the intestine to perform contractions during digestion. Contractions are more effective if this thickness is large [57]. The intestinal diameter expands as the villus density and size as well as the muscle thickness increase, supporting the results in Table 6. These structural improvements in the intestine align with the observed enhancements in growth performance, as a larger surface area for nutrient absorption likely contributed to better feed utilization and overall fish growth. The results of this study are consistent with earlier findings on the positive role of probiotics in terms of intestinal morphology and health [58–63]. For example, a previous study by Ismail et al. [64] demonstrated that the commercial probiotic Lacto Forte had a significant effect on the height and muscular health of Nile tilapia. Similarly, *B. subtilis WB60* and *L. lactis* positively affect Nile tilapia villus height and intestinal muscular thickness [30, 31].

The results of this study on body composition analysis revealed that the protein and MCs of the fish fed probiotics were significantly greater than those of the control fish. However, there was no significant difference in the crude lipid and ash contents among the groups. Similar results were reported by Hassaan et al. [46], who reported that *B. licheniformis* feed supplementation increased the CP, lipid, and dry matter contents but had no significant effect on the ash content of Nile tilapia. Similarly, the application of *Bacillus* spp. in rainbow trout feed positively affected the protein content of the fish compared with the control group [65]. A study by Opiyo et al. [53] reported similar results, where the inclusion of probiotics, including *B. subtilis*, significantly increased the protein content but not the crude lipid content. The dietary supplementation of fish with probiotics improved their growth, and the quality of the fish flesh changed to leaner and rich in proteins. In fact, as many studies have shown, probiotics secrete enzymes, including proteases, which increase the ability of fish to efficiently digest protein compounds and even to break down proteins into monomers and free amino acids [66–69]. These changes in body composition may be profitable from the viewpoints of both aquaculture producers and consumers, and a healthier product will have better market value.

Hematological parameters are very important in the study of the feed quality and general health of fish. The parameters most commonly influenced by dietary supplements are Hb and hematocrit levels [46]. In this study, probiotic supplementation clearly influenced the hematologic parameters of red tilapia. There were significant increases in the RBC count and Hb and hematocrit concentrations in the probiotic-treated groups, especially in the Pd3 group, which presented better values of those parameters. Significant differences were also observed in the MCHC, monocyte count, and platelet count. Similar results were reported by Hassaan et al. [46], who reported that 0.48 × 10^6^ colony-forming unit (CFU) g^−1^*B. licheniformis* significantly positively impacted the Hb, hematocrit, RBCs, and WBCs of Nile tilapia. Similarly, the 0.08% concentration of *B. licheniformis* in the Nile tilapia diet resulted in higher MCHC values than did the low-concentration groups and the control group [39]. These results do not corroborate those of Telli et al. [70], who reported a significant decrease in RBC count and hematocrit concentration in groups supplemented with *B. subtilis* in Nile tilapia. According to the latter author, the reduction in these blood parameters is linked to the reduction in stress in fish receiving probiotics. However, the results of that study are similar to those of the present study with respect to the platelet (thrombocyte) count. Probiotics are described as having immunostimulant properties and are said to promote the multiplication of blood cells. This phenomenon has already been confirmed in humans in the production of red and white cells [71] and in fish, such as *Rhamdia quelen* [72]. With such features, the fish are then well equipped with immunity and oxygen transport, two important functions that the blood plays, owing to significantly improved blood parameters induced by probiotics [73]. These enhancements point to a better immune response and, in general, a better health status of the fish, which is crucial for resistance to disease and survival in aquaculture environments [39, 72].

Microbial analysis revealed an increased count of total viable and beneficial bacteria, such as *Bacillus* spp., in fish fed probiotics and a reduced number of pathogenic bacteria, such as *Vibrio* spp. Similarly, Deng et al. [74] demonstrated that *B. subtilis* supplementation in the diet resulted in a significant increase in the abundance of mainly beneficial bacteria in the gut of Nile tilapia. Another study by Galagarza et al. [75] revealed that the administration of *B. subtilis* in the form of endospores in the diet resulted in a significant increase in the stability of the intestinal ecosystem and microbial diversity of Nile tilapia. Furthermore, a study by Haraz et al. [50] revealed that the use of probiotics in biofloc systems led to an increase in the total number of bacteria in the intestinal tract of Nile tilapia. An analysis of DGGE profiles by Tachibana et al. [39] revealed that *B. subtilis* and *B. licheniformis* supplementation had favorable effects on microbial richness and habitability, increasing the number of potentially beneficial phyla (probiotics) and decreasing the number of potentially harmful phyla in the Nile tilapia gut. On the other hand, in a study by Hassaan et al. [76], *B. subtilis* given as a dietary supplement to Nile tilapia caused a decrease in the overall number of bacteria in the fish gut. Fish are poikilothermic, meaning that one of the factors affecting their gut microbiota is temperature. The findings of this study demonstrated that the water parameters did not change considerably, and as a result, there is an abundance of *Bacillus* because, in addition to being a component of the supplement, they have adapted to their environment. These bacteria subsequently permit the growth of additional identical bacteria, while potentially harmful bacteria are removed through spatial exclusion and possibly other strategies [65]. The shift in the microbial population is, therefore, advantageous because it helps in outcompeting pathogenic microbes that reduce the occurrence of diseases and provide a healthy gut environment [77–80]. The findings regarding intestinal microbiota bolster the findings regarding growth parameters, as beneficial bacteria are crucial for the digestion of nutrients, as they produce digestive enzymes and enhance the host intestine's digestive morphology [27, 81]. These findings highlight the role of probiotics in the modulation of the gut microbiota for health and productivity maintenance in aquaculture species.

## 5. Conclusion

The present study highlighted the great potential of probiotics in general, especially Enterotrophotic, which contains *B. cereus* and *A. nicotianae*, to improve the growth performance, intestinal health, body biochemical composition, hematological parameters, and microbial balance of red tilapia intestines and culture water. This probiotic did not negatively affect water quality. Among the tested dosages, fish fed Pd3 (40 ml/kg) performed best, with maximum weight gain, SGRs and feed efficiency. Additionally, fish fed Pd3 exhibited improved intestinal morphology and higher protein content, further supporting the role of probiotics in enhancing nutrient absorption and overall health. This study highlighted the role of probiotics at adequate dosages in promoting better digestion and absorption of nutrients. The study results further highlighted that probiotic supplementation resulted in a much healthier gut microbiota by increasing the number of beneficial bacteria and reducing potentially pathogenic species, thus contributing to fish health and disease resistance. These results further support that ensuring fish health and productivity with the least possible environmental damage is achieved by using probiotics as potential environmentally friendly and sustainable substitutes for antibiotics in aquaculture.

## Figures and Tables

**Table 1:** Ingredients and proximate analysis of the basal diet used to feed red tilapia fingerlings.

Ingredients	Fish meal	Soybean meal	Rice bran	Wheat flour	Cassava flour	Multivitamin and minerals*⁣*^*∗*^	Sunflower oil
(g/kg)	502	305	46	77	20	15	35
Proximate analysis	Crude protein (%)	Crude lipid (%)	Moisture (%)	Ash (%)	Crude fiber (%)	Total carbohydrates (%)	Gross energy (kcal/100 g)
(%)*⁣*^*∗∗*^	46.28 ± 1.16	9.77 ± 0.28	5.50 ± 0.30	11.81 ± 0.90	7.22 ± 0.33	19.42 ± 0.11	350.73 ± 0.21

*Note:* Fish meal was made by grinding the dried fish available on a local market; rice bran and soybean meal were purchased from a local market; wheat flour (Aashirvaad Superior MP Atta) was produced by Uttam Agro Foods Pvt. Ltd, India; cassava flour was produced by Kokos Natural, India; sunflower oil (Fortune) was produced by Adani Wilmar, India; multivitamin and mineral caps (Becadexamin) were produced by GlaxoSmithKline Pharmaceuticals Ltd, India.

*⁣* ^*∗*^Each multivitamin and mineral capsule contains vitamin A (as vitamin A concentrate oil [IP]) at 5000 IU, vitamin D3 (cholecalciferol [IP]) at 400 IU, vitamin E (tocopheryl acetate [IP]) at 15 mg, vitamin B1 (IP) at 5 mg, vitamin B2 (IP) at 5 mg, nicotinamide (IP) at 45 mg, D-panthenol (IP) at 5 mg, vitamin B6 (IP) at 75 mg, folic acid (IP) at 1000 mcg, vitamin B12 (IP) at 5 mcg, diclofenac calcium phosphate (IP) at 70 mg, copper sulfate pentahydrate (BP) at 0.1 mg, manganese sulfate monohydrate (BP) at 28.01 mg, zinc sulfate monophosphate (IP) at 28.7 mg (equivalent to 10.4 mg of elemental zinc), potassium iodide (IP) at 0.025 mg, light magnesium oxide (IP) at 0.15 mg.

*⁣* ^*∗∗*^Values are expressed as the means ± standard deviations.

**Table 2:** Rearing water parameters of red tilapia fed different dosages of probiotics.

Parameters	Pd0	Pd1	Pd2	Pd3	*p*-Value
Temperature (°C)	27.64 ± 0.40	27.66 + −0.39	27.64 ± 0.38	27.64 ± 0.39	0.38
pH	7.43 ± 0.20	7.45 ± 0.19	7.43 ± 0.22	7.45 ± 0.21	0.91
Salinity (g/L)	0 ± 00.00	0 ± 00.00	0 ± 00.00	0 ± 00.00	—
Alkalinity (mg/L)	36.66 ± 13.20	44.58 ± 22.50	62.08 ± 35.06	58.33 ± 37.00	0.12
Carbonate (mg/L)	0 ± 00.00	0 ± 00.00	0 ± 00.00	0 ± 00.00	—
Bicarbonate (mg/L)	35.83 ± 11.83	44.58 ± 22.50	62.08 ± 35.06	58.33 ± 37.00	0.10
Hydroxide (mg/L)	0 ± 00.00	0 ± 00.00	0 ± 00.00	0 ± 00.00	—
Total hardness (mg/L)	31.25 ± 11.30	31.25 ± 11.30	31.25 ± 11.30	31.25 ± 11.30	1.00
Calcium (mg/L)	10 ± 00	10 ± 00	10 ± 00	10 ± 00	—
Magnesium (mg/L)	21.25 ± 11.30	21.25 ± 11.30	21.25 ± 11.30	21.25 ± 11.30	1.00
Ammonia (mg/L)	0.048 ± 0.03	0.042 ± 0.03	0.143 ± 0.33	0.031 ± 0.02	0.34
Nitrite (mg/L)	0.041 ± 0.04	0.045 ± 0.04	0.062 ± 0.06	0.082 ± 0.08	0.32
Sulfide (mg/L)	BDL	BDL	BDL	BDL	—
DO (mg/L)	6.38 ± 1.19	6.48 ± 0.70	6.16 ± 0.80	6.40 ± 1.06	0.87

*Note:* The values are presented as the means ± SDs.

Abbreviation: DO, dissolved oxygen.

**Table 3:** Growth, feed utilization, and survival parameters associated with different treatments.

Parameters	Pd0	Pd1	Pd2	Pd3	*p*-Value
IBW (g/fish)	2.12 ± 0.29	2.01 ± 0.27	2.02 ± 0.19	2.04 ± 0.26	0.10
FBW (g/fish)	28.71 ± 3.74^a^	34.46 ± 6.84^b^	38.64 ± 7.66^b,c^	42.91 ± 8.26^c^	0.00
NWG (g/fish)	26.54 ± 0.73^a^	32.44 ± 1.20^b^	36.60 ± 1.35^b^	40.97 ± 1.60^c^	0.00
PWG (%)	1232.12 ± 45.63^a^	1652.05 ± 16.69^b^	1807.74 ± 122.03^c^	2142.44 ± 138.24^d^	0.00
ADWG (g/fish/day)	0.32 ± 0.01^a^	0.39 ± 0.01^b^	0.43 ± 0.02^c^	0.49 ± 0.02^d^	0.00
SGR (%/fish/day)	3.08 ± 0.04^a^	3.41 ± 0.01^b^	3.51 ± 0.08^b^	3.70 ± 0.07^c^	0.00
FCR	2.92 ± 0.08^a^	2.39 ± 0.09^b^	2.12 ± 0.08^c^	1.89 ± 0.07^d^	0.00
FCE	0.34 ± 0.005^a^	0.42 ± 0.017^b^	0.47 ± 0.015^c^	0.53 ± 0.02^d^	0.00
Survival rate (%)	72.22 ± 4.81	77.78 ± 4.81	80.56 ± 4.81	88.89 ± 4.81	0.33

*Note:* The values are presented as the means ± SDs. Values with the same superscript letter in each row are not significantly different.

Abbreviations: ADWG, average daily weight gain; FBW, final body weight; FCE, feed conversion efficiency; FCR, feed conversion ratio; IBW, initial body weight; NWG, net weight gain; PWG, percentage weight gain; SGR, specific growth rate.

**Table 4:** Effects of diet on the proximate composition of red tilapia muscle.

Parameters	Pd0	Pd1	Pd2	Pd3	*p*-Value
Moisture (%)	75.13 ± 1.63^a^	74.12 ± 0.73^a,b^	73.79 ± 0.84^a,b^	72.40 ± 1.73^b^	0.04
Crude protein (%)	78.37 ± 3.71^a^	83.66 ± 0.58^a,b^	85.40 ± 2.08^b^	85.79 ± 1.75^b^	0.01
Crude lipid (%)	2.54 ± 0.28	2.03 ± 0.71	2.16 ± 0.31	2.85 ± 0.42	0.20
Ash (%)	4.35 ± 0.72	4.97 ± 0.18	4.40 ± 0.75	3.85 ± 1.23	0.46

*Note:* The values are presented as the means ± SDs. Values with the same superscript letter in each row are not significantly different.

**Table 5:** Hematological parameters of red tilapia fed probiotics at different dosages.

Parameters	Pd0	Pd1	Pd2	Pd3	*p*-Value
Hb (g/dL)	7.20 ± 0.20^a^	8.56 ± 0.45^b^	8.60 ± 0.36^b^	8.56 ± 0.40^b^	0.004
PCV (Hct) (%)	26.93 ± 0.92^a^	32.66 ± 3.00^b^	31.80 ± 0.72^a,b^	34.33 ± 2.60^b^	0.012
RBC (×10^6^/mm^3^)	2.62 ± 0.06	2.89 ± 0.34	2.77 ± 0.15	2.91 ± 0.14	0.356
MCV (fL)	166.33 ± 11.89	174.73 ± 15.10	170.13 ± 0.57	180.63 ± 0.85	0.361
MCH (pg)	44.30 ± 2.55	46.10 ± 5.59	49.03 ± 3.33	44.56 ± 1.66	0.405
MCHC (g/dL)	26.53 ± 0.75^a,b^	26.33 ± 0.96^a,b^	26.56 ± 0.05^a^	24.53 ± 0.94^b^	0.034
RDW (%)	9.96 ± 0.68	12.40 ± 2.30	10.30 ± 0.26	12.10 ± 0.45	0.093
WBC (×10^3^/mm^3^)	2.40 ± 0.10	2.40 ± 0.36	2.73 ± 0.11	2.33 ± 0.20	0.194
Neutrophils (%)	12.96 ± 11.63	4.63 ± 3.83	2.28 ± 0.50	1.66 ± 1.20	0.172
Lymphocytes (%)	78.79 ± 11.19	77.36 ± 7.04	82.10 ± 2.62	92.57 ± 6.20	0.127
Monocytes (%)	6.47 ± 0.25^a,b^	13.46 ± 0.51^a^	13.83 ± 3.97^a^	4.77 ± 4.20^b^	0.009
Eosinophils (%)	1.77 ± 0.91	4.53 ± 3.70	1.78 ± 1.33	0.99 ± 0.87	0.245
Basophils (%)	0 ± 00	0 ± 00	0 ± 00	0 ± 00	
Platelet count (×10^3^/mm^3^)	3.33 ± 0.57	5.66 ± 1.15	7.33 ± 1.15	9.66 ± 4.50	0.063
MPV (fL)	7.93 ± 0.70	10.30 ± 1.08	10.53 ± 1.43	9.83 ± 3.11	0.356

*Note:* The values are presented as the means ± SDs. Values with the same superscript letter in each ow are not significantly different.

Abbreviations: Hb, hemoglobin; MCH, mean corpuscular hemoglobin; MCHC, mean corpuscular hemoglobin concentration; MCV, mean corpuscular volume; MPV, mean platelet volume; PCV, packed cell volume; RBC, red blood cell; RDW, red cell distribution width; WBC, white blood cell.

**Table 6:** Effects of probiotics on the intestinal histomorphology of red tilapia

Parameter	Pd0	Pd1	Pd2	Pd3	*p*-Value
Villi height (µm)	249.57 ± 47.67^a^	303.50 ± 44.35^a^	525.90 ± 56.42^b^	577.78 ± 84.08^b^	0.000
Villi width (µm)	45.96 ± 7.92^a^	51.0 ± 11.77^a^	68.55 ± 15.35^b^	72.61 ± 11.53^b^	0.000
Muscular thickness (µm)	68.04 ± 18.09^a^	55.64 ± 7.19^a^	83.86 ± 18.92^a,b^	101.83 ± 17.48^b^	0.000
Section diameter (µm)	1538.29 ± 130.17^a^	1669.22 ± 133.57^a,b^	1701.09 ± 103.32^ab^	1705.26 ± 135.07^b^	0.035

*Note:* The values are presented as the means ± SDs. Values with the same superscript letter in each row are not significantly different.

## Data Availability

The data that support the findings of this study are available from the corresponding author upon reasonable request.
